# Lily Pollen Tubes Pulse According to a Simple Spatial Oscillator

**DOI:** 10.1038/s41598-018-30635-y

**Published:** 2018-08-14

**Authors:** Milenka Van Hemelryck, Roberto Bernal, Yaroslav Ispolatov, Jacques Dumais

**Affiliations:** 1 Departamento de Física, Universidad de Santiago de Chile, Santiago, 9170124 Chile; 2 Facultad de Ingeniería y Ciencias, Universidad Adolfo Ibáñez, Viña del Mar, Region V Chile

## Abstract

Polar growth is a fundamental mode of cell morphogenesis observed in nearly all major groups of organisms. Among polarly growing cells, the angiosperm pollen tubes have emerged as powerful experimental systems in large part because of their oscillatory growth, which provides a window into the network of interactions regulating morphogenesis. Empirical studies of oscillatory pollen tubes have sought to uncover the temporal sequence of cellular and molecular events that constitutes an oscillatory cycle. Here we show that in lily pollen tubes the distance or wavelength (*λ* = 6.3 ± 1.7 *μ*m) over which an oscillatory cycle unfolds is more robust than the period of oscillation (*τ* = 39.1 ± 17.6 s) (n = 159 cells). Moreover, the oscillatory cycle is divided into slow and fast phases, with each phase unfolding over precisely one half of the wavelength. Using these observations, we show that a simple *spatial* bi-oscillator predicts the most common modes of oscillation observed in pollen tubes. These results call into question the traditional view of pollen tube morphogenesis as a temporal succession of cellular events. Space, not time, may be the most natural metric to inteprete the morphogenetic dynamics of these cells.

## Introduction

Growth is a fundamental attribute of the living cell. Although this process has been studied extensively, and the mechanistic basis for growth in specific cell types is now better understood, there are still few models connecting, quantitatively, specific molecular processes and observed growth dynamics of cells^1–3^. One important growth process where substantial progress has been made is tip growth morphogenesis as seen in root hairs, pollen tubes, and fungal hyphae^4–6^. Recent models of polar growth have tackled how polarity is achieved and maintained^7,8^, how growth may be guided in space^2^, and how the shape of the growing tip is specified at the subcellular level^1,9–11^.

The discovery of oscillatory pollen tube growth more than two decades ago has motivated researchers to exploit this phenomenon to piece together the different feedback interactions that control polar growth^12–16^. Although there is still a debate as to whether rhythmic fluctuations in the velocity are part of the normal *in vivo* morphogenesis of pollen tubes^17^, there cannot be any doubt that oscillatory growth is a powerful tool to analyze the molecular and biophysical control of cell morphogenesis. The opportunity offered by regular growth oscillations was cleverly exploited to order cellular events temporally through cross-correlation analysis^18^. In a sense, oscillatory growth is a ready-made pulse-chase experiment whereby slight cycle-to-cycle fluctuations in the elongation rate and other cellular variables can be “chased” through various downstream cellular processes giving a clear picture of how cytological events succeed each others in time^16,18–22^.

Many measurable cytological changes associate with oscillatory growth, including changes in calcium and proton concentrations^13,23–25^, ions influxes across the plasma membrane^18,26,27^, apical cell wall thickness^28,29^, and the concentrations of enzymes in the cytoplasm^30,31^. An interesting pattern is now emerging from these analyses: processes associated with actin polymerisation and secretion^25,30,32^ *precede* the maximum in elongation rate, as do wall-based processes such as the accumulation of wall material at the tip^12,28^ and the concentration of pectin methyl-esterase (PME)^28^. In contrast, the cytosolic concentration of many ions such as calcium and ion influxes across the membrane^13,26,27^ *follow* the maximum in elongation rate. This temporal ordering of events suggests the following causal sequence: (*i*) the activitation of actin polymerisation and secretion at the tip causes an increase in wall deposition (pectins) and the remodelling of wall pectins by the enzyme PME; (*ii*) the wall remodelling softens the tip leading to an accelerated elongation rate; (*iii*) finally, a rapidly elongating tip promotes the flux of ions into the cytosol perhaps through the action of stretch-activated ion channels^33^. It is not clear, however, what other feedback mechanisms maintain the stability of the morphogenetic process. Moreover, oscillations have also been reported in non-growing pollen tubes suggesting that more than one oscillator might be present in these cells^16,34,35^.

Recently, we presented a detailed kinematic analysis of oscillatory growth based on a large dataset of time-lapse sequences of lily pollen tubes^36^. Following inspection of this dataset, it became clear that the current focus on the period of oscillation and related attempts to order cellular events temporally may in fact be neglecting the most salient dynamical feature of oscillating pollen tubes - the surprisingly stable distance or wavelength over which each oscillatory cycle unfolds. In what follows, we show that oscillatory growth is best interpreted as a spatial “stepping” process rather than a clock-like process.

## Results

The results below were obtained with image analysis tools designed to track the contour of pollen tubes growing under standard culture conditions (Methods). A total of 159 lily pollen tubes are included in this analysis. Unless otherwise indicated, images were acquired every 2 s with a spatial resolution of 0.13 *μ*m per pixel. Complementary observations were also performed on *Petunia* pollen tubes.

### Dynamics of oscillatory growth

**Figure 1:**
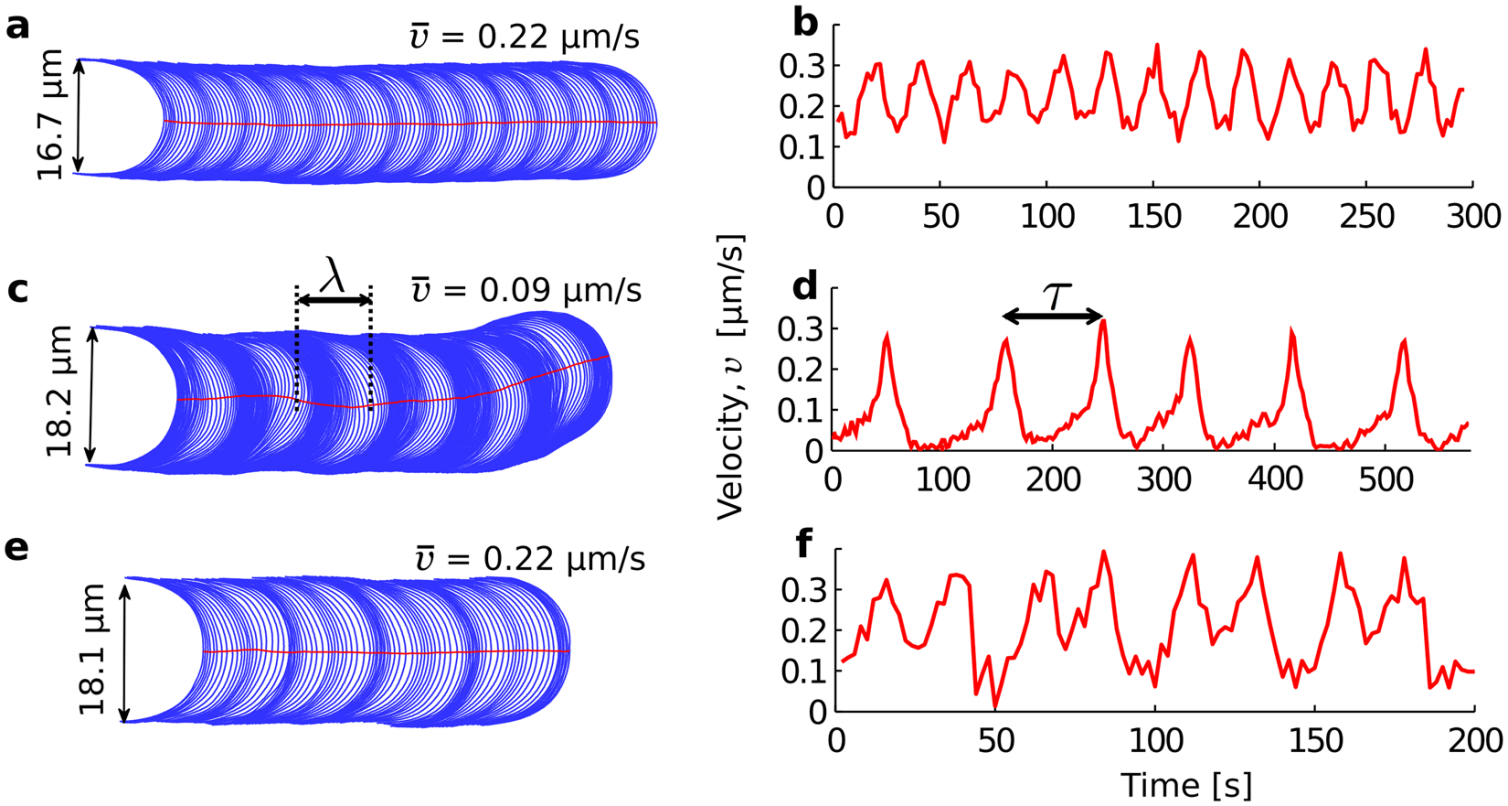
Modes of oscillatory growth in lily pollen tubes. (**a**, **c** and **e**) Cell outlines captured every two seconds. (**b**, **d** and **f**) The corresponding pole velocity, which can be symmetrical (**b**), pulsing (**d**), and even doubly-periodic (**f**). The wavelength (*λ*) and period (*τ*) are defined graphically in (**c** and **d**).

**Figure 2:**
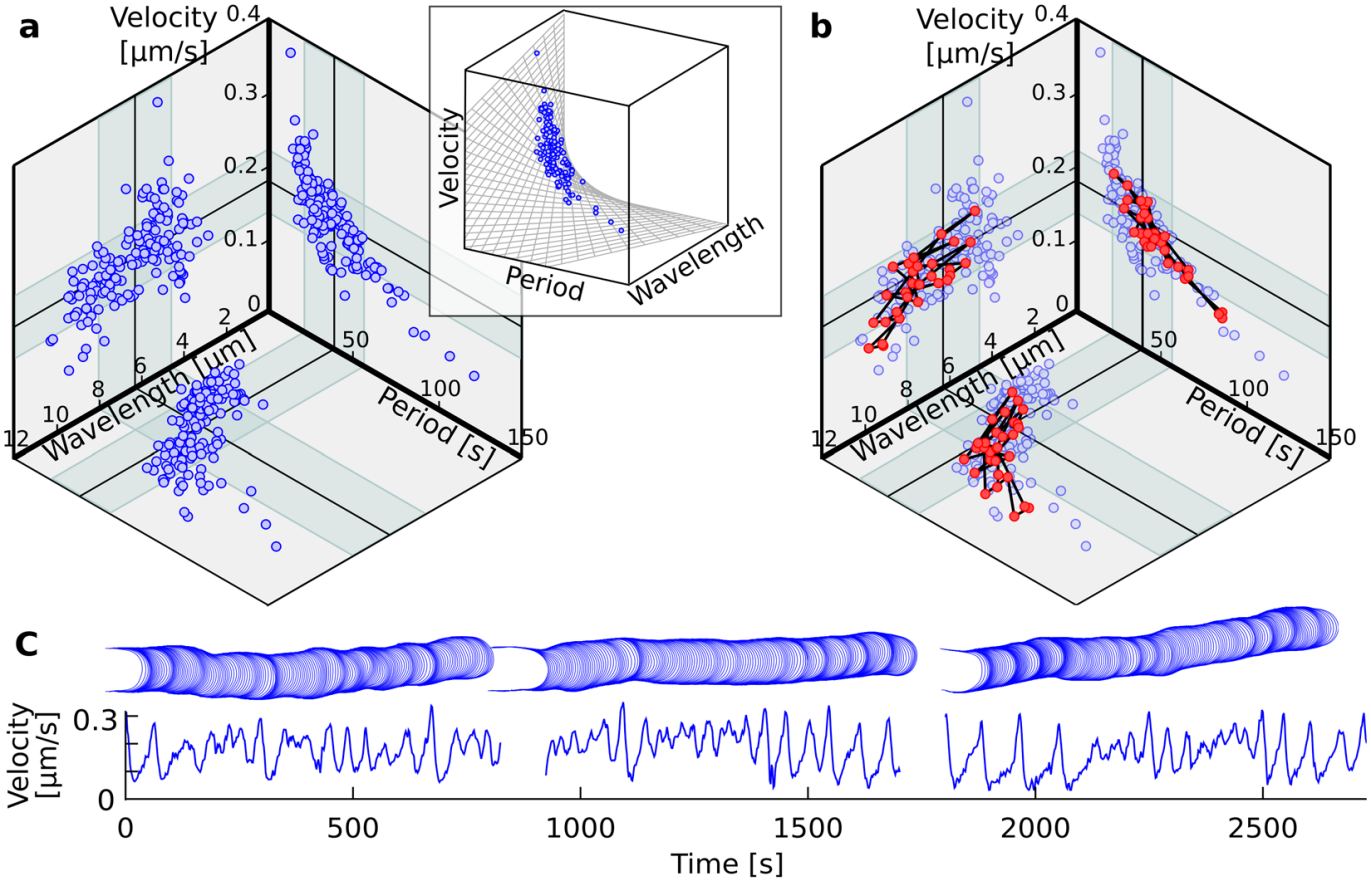
Relationship between wavelength, period, and velocity in lily pollen tubes. (**a**) Wavelength, period, and velocity in a population of 159 pollen tubes. Each point corresponds to averages for a single pollen tube recorded for at least four cycles (2.1 min). The results are presented as projections of the points $({\bar{\lambda }}_{k},{\bar{\tau }}_{k},{\bar{v}}_{k})$ onto the planes $(\bar{\lambda },\bar{\tau },0)$, $(\bar{\lambda },0,\bar{v})$, and $(0,\bar{\tau },\bar{v})$. Inset: 3D representation of the triplets on the surface *v* = *λ*/*τ* where they are confined. (**b**) The trajectory of one pollen tube recorded for 27 minutes (red circles) is overlaid onto the cell averages (blue circles). (**c**) The full time series for the cell plotted in (**b**). Of the more than 40 minutes of recording, 27 min showed a sufficiently clear oscillation to be included in the analysis (Methods).

Although pollen tubes can grow at a steady velocity^36^, a majority of lily pollen tubes show some degree of oscillation (Fig. 1). The most frequent type of oscillation (85%) is fairly symmetrical with a triangular waveform (Fig. 1a,b). The second most common type of oscillation (13%) is best described as pulsing or bursting because relatively long periods of low elongation rate give rise to short bursts of high elongation rate (Fig. 1c,d). In some rare cases (2%), cells achieve complex, yet reproducible, oscillatory patterns (Fig. 1e,f). Finally, we note that cells can shift relatively quickly between these different modes of oscillation, often over a single oscillation cycle (Fig. 2c).

The dynamics of oscillatory cells can be characterised by three variables: the time-dependent tip velocity (*v*(*t*)), as well as the period (*τ*) and wavelength (*λ*) of each oscillation. The last two variables are measured independently by tracking the displacement of the pollen tube in time-lapse sequences and identifying the start and end points of every oscillatory cycle (Fig. 1c,d, see Methods for details). The point of maximum velocity is the easiest feature to identify on successive cycles and was therefore selected to mark the beginning and end of each cycle.

We define the mean velocity of cell *k* as: ${\bar{v}}_{k}={\bar{\lambda }}_{k}/{\bar{\tau }}_{k}$, where ${\bar{\lambda }}_{k}$ and ${\bar{\tau }}_{k}$ are the mean wavelength and mean period recorded for the cell. Since this equation establishes a formal relation between the three kinematic variables, the plotting of any two variables would be sufficient to describe fully the kinematics. Yet, for pollen tubes, it is not clear which of the three variables should be eliminated or interpreted in terms of the other two. This choice should be based on a clear understanding of how these variables are controlled at the cellular or molecular level. Without this information, we favor a representation that considers the tri-dimensional triplets $({\bar{\lambda }}_{k},{\bar{\tau }}_{k},{\bar{v}}_{k})$ and their projections onto three orthogonal planes to facilitate the visualisation of the relation between every pairs of variables (Fig. 2a).

Inspection of the $\bar{\lambda }-\bar{\tau }-\bar{v}$ state space reveals some interesting features. At the population level, the wavelength fluctuates over a limited range of 2.8 to 10.2 *μ*m while the period ranges from 14.0 to 122.4 s (Table 1). Correlation analyses between pairs of variables give the following results: $R({\bar{v}}_{k},{\bar{\tau }}_{k})=-\,0.70$; $R({\bar{v}}_{k},{\bar{\lambda }}_{k})=-\,0.27$; and $R({\bar{\lambda }}_{k},{\bar{\tau }}_{k})=0.79$ (Fig. 2a). The strong negative correlation between velocity and period is to be expected if the wavelength is fairly constant. Similarly, a strong positive correlation between wavelength and period is indicative of a velocity fluctuating over a narrow range. Finally, a strong *positive* correlation between wavelength and velocity would have been expected if the period was relatively constant. The weak negative correlation recorded for this pair of variables is thus the first indication that the period may not be the most reliable descriptor of the kinematics of tip growth. According to these population-level observations, the wavelength and velocity are the least fluctuating kinematic variables while the period shows the largest fluctuations (Table 1).

**Table 1:** Statistics of the kinematic variables evaluated at the population level (n = 159 cells).

Variables	Range	Mean	Std (CV)
wavelength (*λ*) [*μ*m]	2.8–10.2	6.3	1.7 (27%)
period (*τ*) [s]	14.0–122.4	39.1	17.6 (45%)
velocity (*v*) [*μ*m/s]	0.08–0.37	0.16	0.04 (24%)

The coefficient of variation (CV) was computed as (std/mean) × 100%.

### The morphogenetic dynamics are ergodic

Before pursuing further our analysis, we turn to a question rarely raised in studies of tip growth morphogenesis but which seems particularly important in the present context: are the dynamics inferred at the population level representative of the dynamics of individual pollen tubes? The evocative trends observed in Fig. 2a were obtained from a large population of cells where each cell is represented by three variables averaged over a fairly short time interval (typically 5 min). It is in that context that the period emerges as the most fluctuating variable (Table 1). Thus, *as a population*, lily pollen tubes seem to have a better control over their wavelength and velocity than over their period. However, this population-level observation may or may not apply to individual cells. For instance, the trends observed in Fig. 2a could reflect the genetic variability of the haploid vegetative nucleus controlling the morphogenesis of pollen tubes. Because of their particular allelic make-up, some tubes could be oscillating with longer or shorter periods but with each tube having a well-defined period. This genetic explanation for the trends in the $\bar{\lambda }-\bar{\tau }-\bar{v}$ space would obviously be quite distinct from an explanation whereby all tubes are equal in morphogenetic potential and all exert poor control over their period of oscillation as compared to their wavelength.

**Figure 3:**
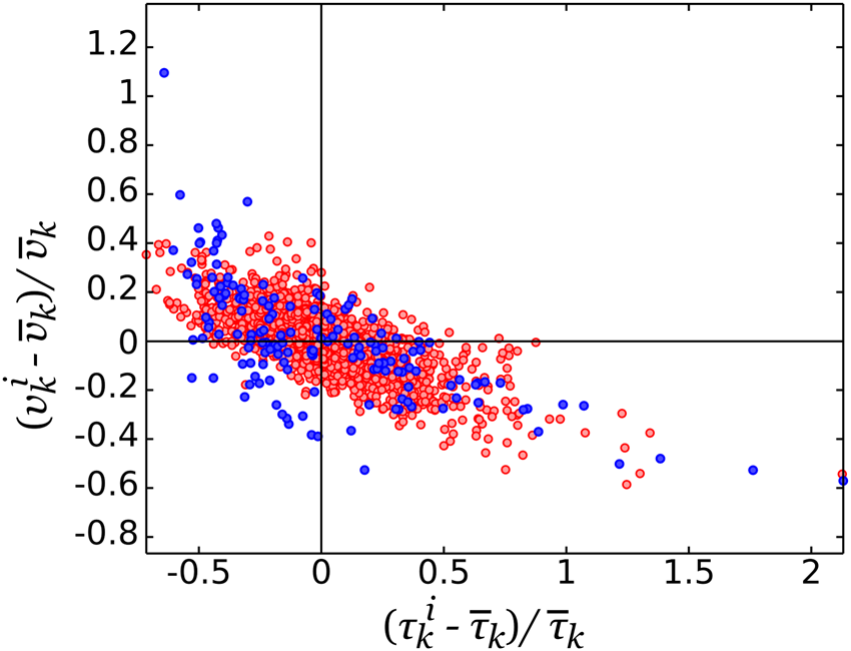
Fluctuation analysis of the velocity and period. The fluctuations computed at the cell level (red dots), where ${v}_{k}^{i}$ and ${\tau }_{k}^{i}$ correspond to the velocity and period of cycle *i* in cell *k*, are overlaid with the population-level fluctuations in these variables (blue dots).

To address this question, we tracked several pollen tubes over a long period of time to determine whether the same behaviour as that reported at the population level would be observed. Interestingly, a single cell tracked over 27 min can reproduce nearly the full range of values and the same trends as those recorded for the population (Fig. 2b,c). We also looked more specifically at the fluctuations in the velocity-period plane (Fig. 3). The range of fluctuations at the cell level and population level can be superposed precisely and thus reveals equivalent dynamics at both levels. We conclude that the morphogenetic dynamics are ergodic in the sense that a single cell, if tracked over a sufficiently long period of time, can reproduce the same dynamics as that inferred by analysing a large population of cells, with each cell tracked for a much shorter period of time. Since any given pollen has a fix genome and is unlikely to undergo epigenetic changes within the time scale of our experiments, the distributions of $\bar{\lambda }$, $\bar{\tau }$, and $\bar{v}$ must have a physiological basis rather than a genetic one.

### The growth dynamics conserve the wavelength and the spatial symmetry of the velocity distribution

**Figure 4:**
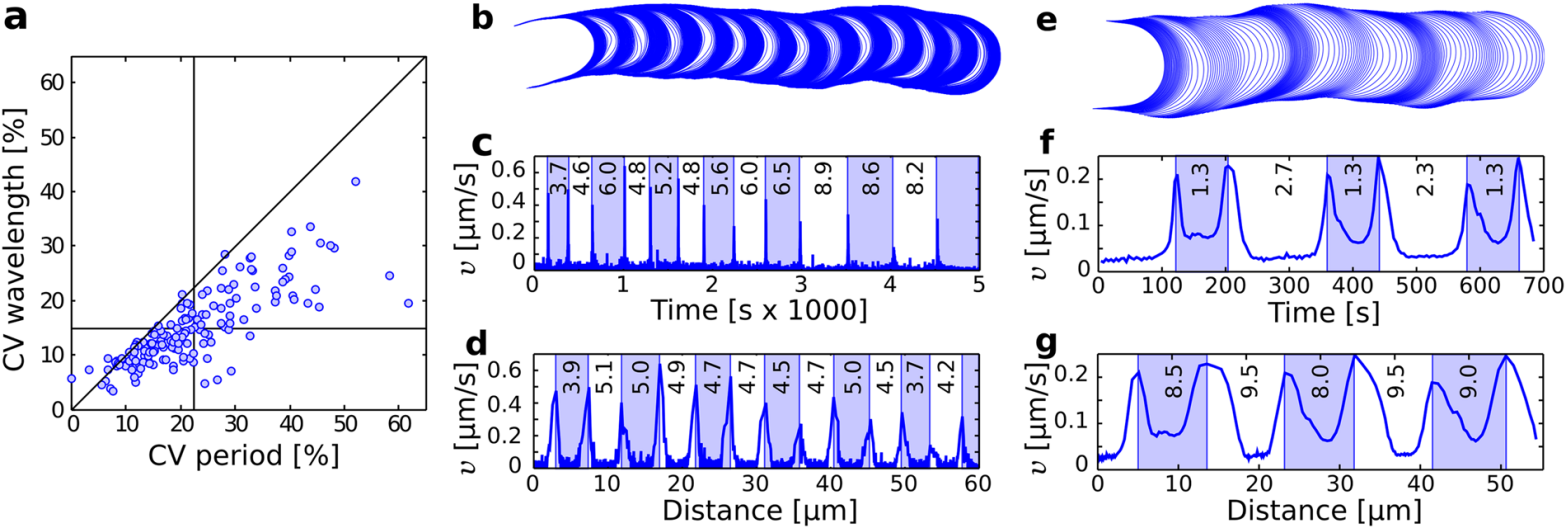
Fluctuation analysis of the period and wavelength. (**a**) Coefficient of variation of the wavelength and period recorded *within* individual cells. The period is systematically more variable ($\overline{{\rm{CV}}}(\tau )=22 \%$) than the wavelength ($\overline{{\rm{CV}}}(\lambda )=15 \%$). (**b**–**d**) A *Petunia* pollen tube showing a steadily increasing period (**c**) while the wavelength remains nearly constant (**d**). (**e**–**g**) Lily pollen tube growing with a clear double period (**f**) but nearly constant wavelength (**g**).

To quantify how the wavelength and period fluctuate within a cell, we used the coefficient of variation ${\rm{CV}}{(x)}_{k}=\frac{{\rm{std}}{(x)}_{k}}{{\rm{mean}}{(x)}_{k}}100 \%$ of variable *x* for pollen tube *k*. For nearly all cells, the wavelength shows a lower internal coefficient of variation than the period (Fig. 4a). Moreover, a detailed error analysis establishes that the greater fluctuation in the period did not arise from some methodological bias in the measurement of these variables (Methods). Yet the most striking illustration of the superiority of the wavelength as a metric for analysing oscillatory growth comes from the observation of oscillatory patterns in specific pollen tubes. For example, the growth dynamics of *Petunia* pollen tubes are characterised by short pulses of growth separated by long intervals of near-zero growth (Fig. 4b–d). The period in those cells can fluctuate widely (Fig. 4c). However, when the velocity is plotted as a function of distance, the oscillatory cycles show a near constant wavelength (Fig. 4d). Another compelling example supporting the superiority of wavelength comes from pollen tubes with “doubly-periodic” elongation rates (Fig. 4e). In such cells, the period can alternate between two distinct values (Fig. 4f) while the wavelength remains surprisingly constant (Fig. 4g). Thus, analyses of oscillatory dynamics at the population level (Fig. 2), at the cell level (Fig. 4a), and in specific cases of more complex oscillatory patterns (Fig. 4b–g) all converge to the same conclusion: the growth dynamics of pollen tubes are characterised by a stable wavelength and a fluctuating period.

**Figure 5:**
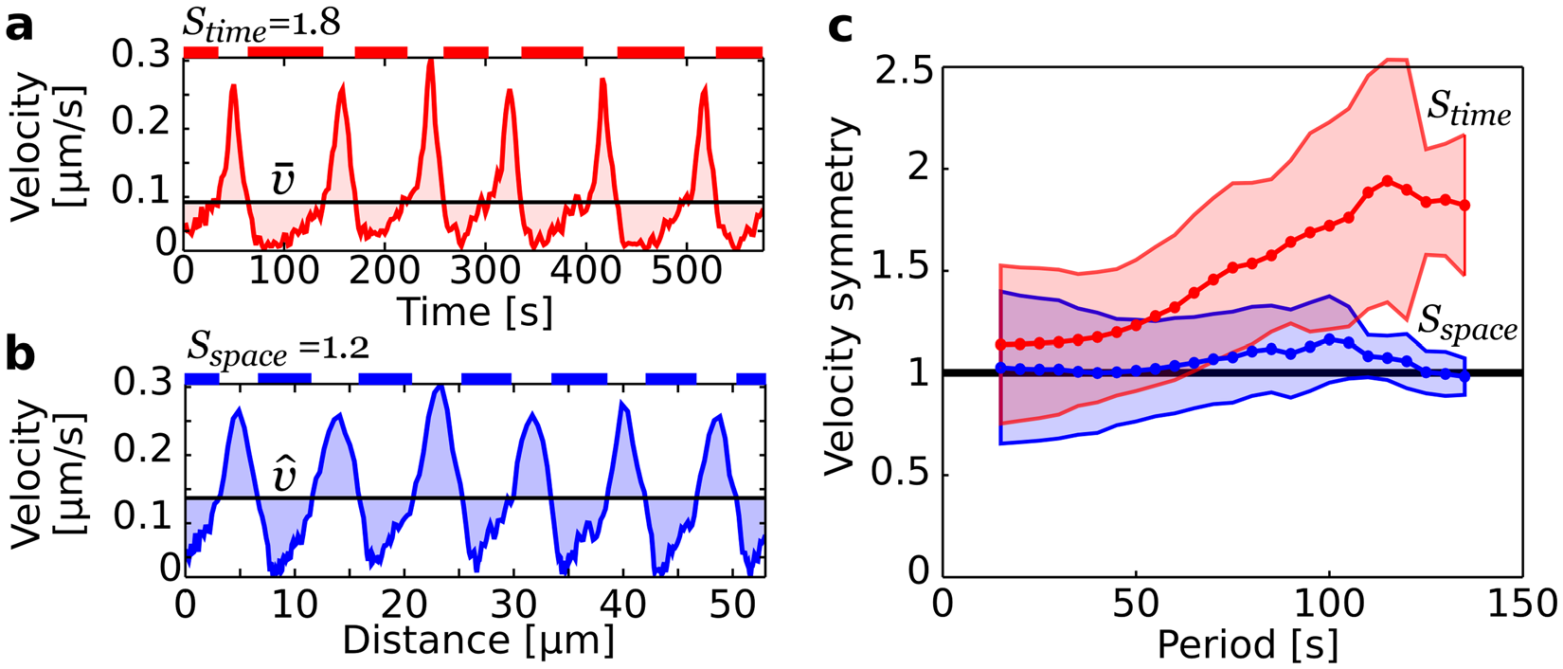
Symmetry in the distribution of velocity. (**a** and **b**) Temporal and spatial evolution of the velocity for the cell illustrated in Fig. 1b. The time and distance spent at low velocities are marked as red and blue line segments in (**a** and **b**). The cell is seen to spend more time at low velocities (**a**), with the net result that nearly equal distances are covered at low and high velocities (**b**). (**c**) Analysis of 2865 cycles recorded in 159 cells for the time spent at low and high velocity (red curve) versus the distance covered at low and high velocity (blue curve). Complete symmetry between low and high velocities is given by a ratio of 1 (black line).

Inspection of the cell contours (Fig. 1) reveals that pollen tubes do more than maintain a relatively constant wavelength, they also appear to divide every oscillatory cycle into symmetrical slow and fast phases, each phase unfolding over a half-wavelength. This feature is most striking when looking at the contours of *Petunia* pollen tubes whose near symmetrical spatial banding of high and low velocities (Fig. 4b) contrasts sharply with the ephemeral nature of their growth bursts (Fig. 4c). In order to establish firmly this conclusion, we quantified the symmetry of the velocity by sampling its distribution both in time and in space using the following ratios:

1 $${S}_{time}=\frac{\{t:v(t) < \bar{v}\}}{\{t:v(t)\ge \bar{v}\}}\,{\rm{and}}\,{S}_{space}=\frac{\{s:v(s) < \hat{v}\}}{\{s:v(s)\ge \hat{v}\}}$$

where $\bar{v}(t)={\int }_{\tau }\,v(t)dt/\tau$ is the mean velocity sampled in time and $\hat{v}(s)={\int }_{\lambda }\,v(s)ds/\lambda$ is the mean velocity sampled in space. The temporal velocity ratio, *S*_*time*_, is the time spent at velocities below the mean velocity divided by the time spent at velocities exceeding the mean velocity (Fig. 5a). The spatial velocity ratio, *S*_*space*_, can be intepreted similarly as the distance traveled at below average velocities divided by the distance traveled at above average velocities (Fig. 5b). Note that both parameters are computed over *complete* cycles.

The velocity ratios were computed for the 2865 cycles available both in the space and time domains (Fig. 5c). The velocity ratio in the space domain maintains a “low-high” symmetry for the entire range of periods observed while the velocity ratio computed in the time domain is always biased towards low velocities, much more so for long periods. Thus, Fig. 5c confirms that cells advance near equal distances during the slow and fast phases of their oscillatory cycles.

### A simple spatial bi-oscillator predicts observed modes of growth

**Figure 6:**
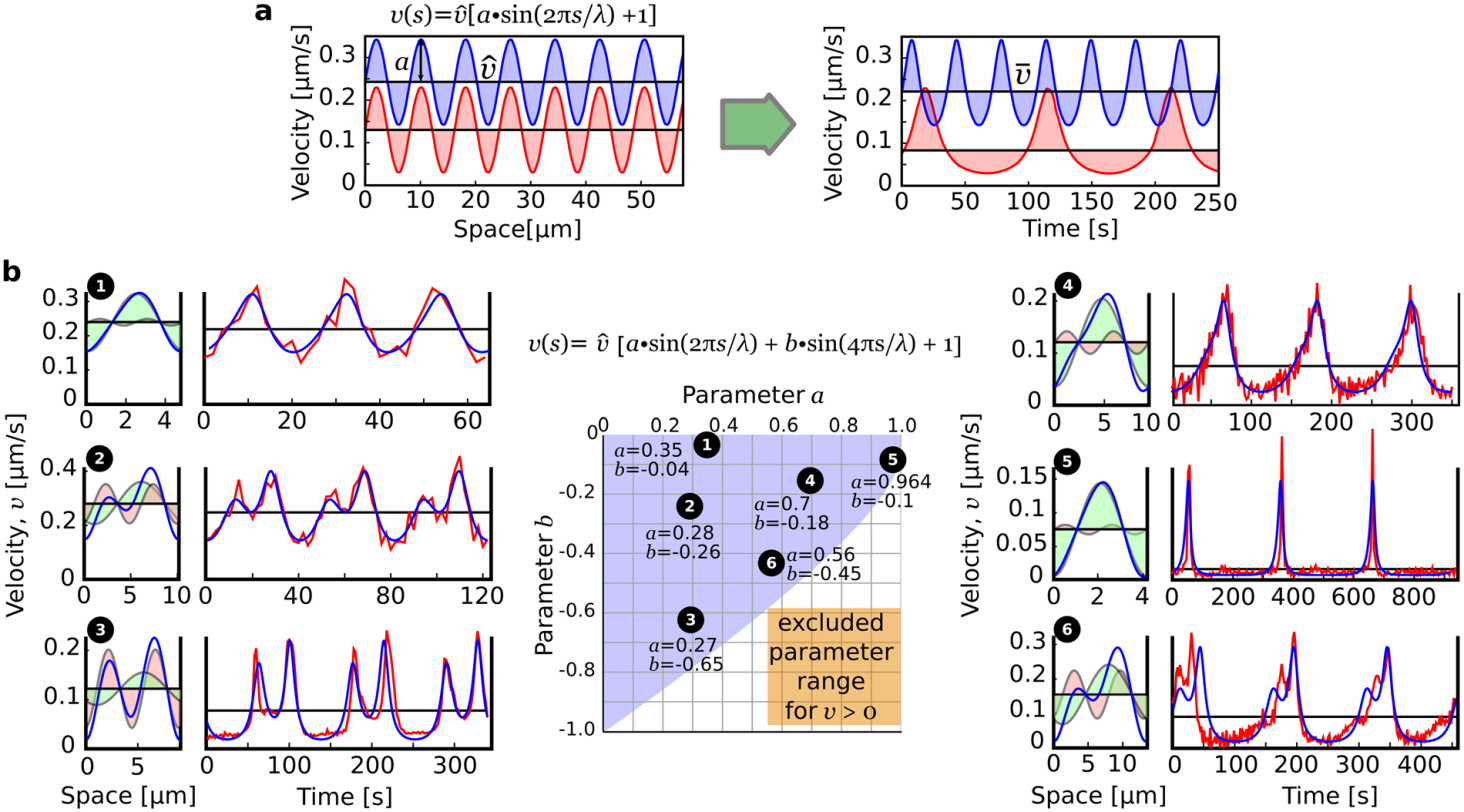
Prediction of observed velocity time series with a bi-oscillator in the space domain. (**a**) Examples of the distortion of the waveform associated with the mapping from the space domain to the time domain. Note that a low mean velocity is associated with a longer period, as seen experimentally. (**b**) Prediction of six waveforms with a simple bi-oscillator equation. For each time series, the left panel shows the two sine components and the resulting waveform in the space domain while the right panel shows a time series of an observed waveform (red curve) and its fit with the bi-oscillator equation (blue curve). The central panel shows where each of the waveforms is located in the *a*–*b* parameter space.

The results of the previous sections were obtained without grouping cells based on their waveform or mode of oscillation; yet these modes are diverse and complex (Fig. 1). Can we therefore assume that all modes of oscillation in lily pollen tubes share the features of a stable wavelength and equal distances traveled at low and high velocities? We explore this question by studying simple spatial oscillators of the form: $v(s)=\hat{v}[a\,\sin (2\pi s/\lambda )+1]$. This functional form has built in a constant wavelength divided into equal phases of low and high velocities. If these velocity functions are projected back in the time domain using the relation $t=\int \,v{(s)}^{-1}ds$, we observe a distortion of the oscillations reminescent of the velocity series recorded in pollen tubes (Fig. 6a), in particular, low mean velocities ($\bar{v}$ or $\hat{v}$) are associated with longer oscillation periods as observed in our dataset (Fig. 2a). In the case of a single sine function, however, the waveform maintains a left-right symmetry while actual cells often show asymmetrical waveforms (e.g. Fig. 1b). To account for this feature, a second in-phase oscillator could be added to get the function: $v(s)=\hat{v}[a\,\sin (2\pi s/\lambda )+b\,\sin (4\pi s/\lambda )+1]$. This equation is arguably the simplest mathematical model that fixes the wavelength, maintains equal displacements at low and high velocities, and allows waveforms with left-right asymmetry (Methods). Despite its simplicity (only the *a* and *b* parameters are not fixed by observations), the “bi-oscillator” equation reproduces accurately a wide spectrum of oscillatory modes commonly observed in pollen tubes (Fig. 6b). These include asymmetric waveforms, double peaks, and the bursting growth seen in *Petunia* (Fig. 6b).

## Discussion

A striking picture emerges from our analysis - much of the perceived complexity of oscillatory tip growth vanishes when the observed time series are interpreted in terms of a velocity specified as a function of distance instead of time. Within the space domain, a two-parameter bi-oscillator accounts for the growth dynamics and the richness of waveforms observed in pollen tubes, including species with very distinct dynamics such as lily and *Petunia*. Although these observations do not point directly to a specific mechanism of polar growth, they do impose strong constraints on any putative mechanism for the morphogenesis these cells. Concretely, future morphogenetic mechanisms must lead to oscillations that:

(i) show greater stability (Fig. 4a) and symmetry (Fig. 5) in the space domain than in the time domain.
(ii) can quickly transit between different modes (Fig. 2b,c).
(iii) include, at the very least, single and double oscillation modes (Fig. 6).

We add that the wavelength and the period of oscillation are unlikely to be under direct cellular control since both are “integrals” over an entire growth cycle. Presumably, these features of the growth dynamics are emergent properties of molecular processes such as the rate of actin polymerisation, the rate of secretion and wall chemistry over which the cell has more direct control. Perhaps then, the wavelength is relatively constant not because it is under direct homeostatic control by the cell but because it is one of the emergent dynamical features better buffered against the noise inherent to the molecular processes controlling morphogenesis. Therefore, much like oscillatory growth itself, the significance of the stable and symmetrical wavelength is not as a newly discovered adaptive feature of pollen growth *in vivo* but rather as one more quantitative tool at our disposal to elucidate the sequence of events controlling morphogenesis.

The greater robustness of the spatial dynamics over the temporal dynamics reported here is not unique to pollen tubes. In fact, a parallel can be drawn between our observations and recent studies of cell division in bacteria^37^. Cell cycle control has been commonly thought of as a timing mechanism. Yet, it is now known that some bacteria, including *E*. *coli*, can divide according to a “adder” mechanism; that is, these cells incorporate a fixed increment of material over each cell cycle^38^. As a corollary to this control mechanism, the duration of the cell cycle (equivalent to the period in our study) must be adjusted by the cell to correct for fluctuations in cell size at birth. In other words, cell length at division seems more important than the duration of the cell cycle. Related observations were made on the branching pattern of *Neurospora* hyphae whose branching density is robust against treatments affecting hyphal growth rate^39^. As a result, the median distance between branches is conversed while the time interval between successive branching events can vary greatly. Although the molecular bases for these processes are likely to be distinct, the broad similarities may underscore the superiority of “measuring” mechanisms over “timing” mechanisms for controlling certain aspects of cell morphogenesis.

Finally, our conclusions call for a re-interpretation of previously published results. First, given the greater stability of the wavelength, it would be informative to report the phase relationships between cellular events and growth in terms of their relative spatial position within the oscillation wavelength rather than their timing of arrival during the oscillation period. Although this change of metric will not alter the sequential ordering of cellular events, it may reveal other invariant features that get blurred when using the more variable period as a metric. Second, a number of earlier studies have reported various experimental treatments of pollen tubes that slow the elongation rate and concomitantly increase the period of oscillation^40–43^. The inverse relation between velocity and period suggests, as for our own dataset, a robust wavelength. Unfortunately, the wavelength of oscillation is rarely reported so it is impossible at this stage to determine the robustness of the dynamical features under various experimental treatments. We hope that our results will motivate future studies to consider both the period and wavelength when analysing the oscillatory dynamics of pollen tubes.

## Methods

### Sample preparation and microscopy

Pollen grains were obtained from asiatic lily cultivars (*Lilium sp*.) purchased at local florists. Pollen grains were germinated in growth medium containing 15 mM MES buffer, 1.6 mM H_3_BO_3_, 0.1 mM CaCl_2_, 0.1 mM KCL, and 7.5% sucrose, adjusted to pH 5.3 with KOH. *Petunia* pollen grain were germinated in growth medium containing 1.6 mM H_3_BO_3_, 1.3 mM Ca(NO_3_)_2_4H_2_O, 1 mM KNO_3_, 0.8 mM MgSO_4_7H_2_O, and 12% sucrose.

For microscopic observations, a square chamber made with dental polymer was afixed to a glass slide. A thin layer of growth medium supplemented with 1% low melting point agarose was deposited inside these chambers. While the agarose was still in a molten state, pollen grains were deposited onto the surface. After approximately 40 minutes, the chamber was filled with growth medium and covered with a cover slip. In this way, pollen tubes grew along the gel-liquid interface rather than in-and-out of the imaging plane, which was ideal for imaging. All cells were imaged through a 40x objective with bright-field microscopy on an inverted microscope (Olympus IX81) equiped with a Thorlabs camera (DCC1545M-GL). The microscope and camera were controlled with the ImageJ Micromanager software. Lily and *Petunia* pollen tubes were imaged at time intervals of 2 seconds (in some case 3 or 4 seconds) and 1 to 5 seconds respectively starting at different times after inoculation. All time-lapse sequences and the velocity time-series used for this manuscript are available upon request (jacques.dumais@uai.cl).

### Image analysis

Image analysis was performed using ImageJ and Matlab^10,36^. Custom Matlab routines were written to extract the cell outlines and compute from them the position of the pole of the pollen tube and the elongation rate. First, a bright-field image sequence was loaded into Matlab. Then approximate contour points on the first image were manually selected. A cubic spline interpolation was used to obtain regularly spaced points around the cell outline. Afterwards, the routine maximised the fit between the splined contour and the cell edges in the image and then tracked the evolution of the cell edge over the entire stack. To this end, the normal vectors were computed for each point of the outline. These normal vectors were then used to take small steps orthogonal to the cell contour in order to predict the position of the next contour.

To determine the pole of the cell, orthogonal paths were drawn from the last cell outline to the first one in such a way that the distances to the next outline were minimised. The growth axis (pole) was defined as the longest trajectory between the first and the last cell outline. The tip velocity (rate of elongation) was computed based on the displacement of a five-point neighbourhood around the pole of the cell. The width of the cell was measured by averaging the shortest distance between opposing sides of the cell at fixed distances from the pole.

**Figure 7:**
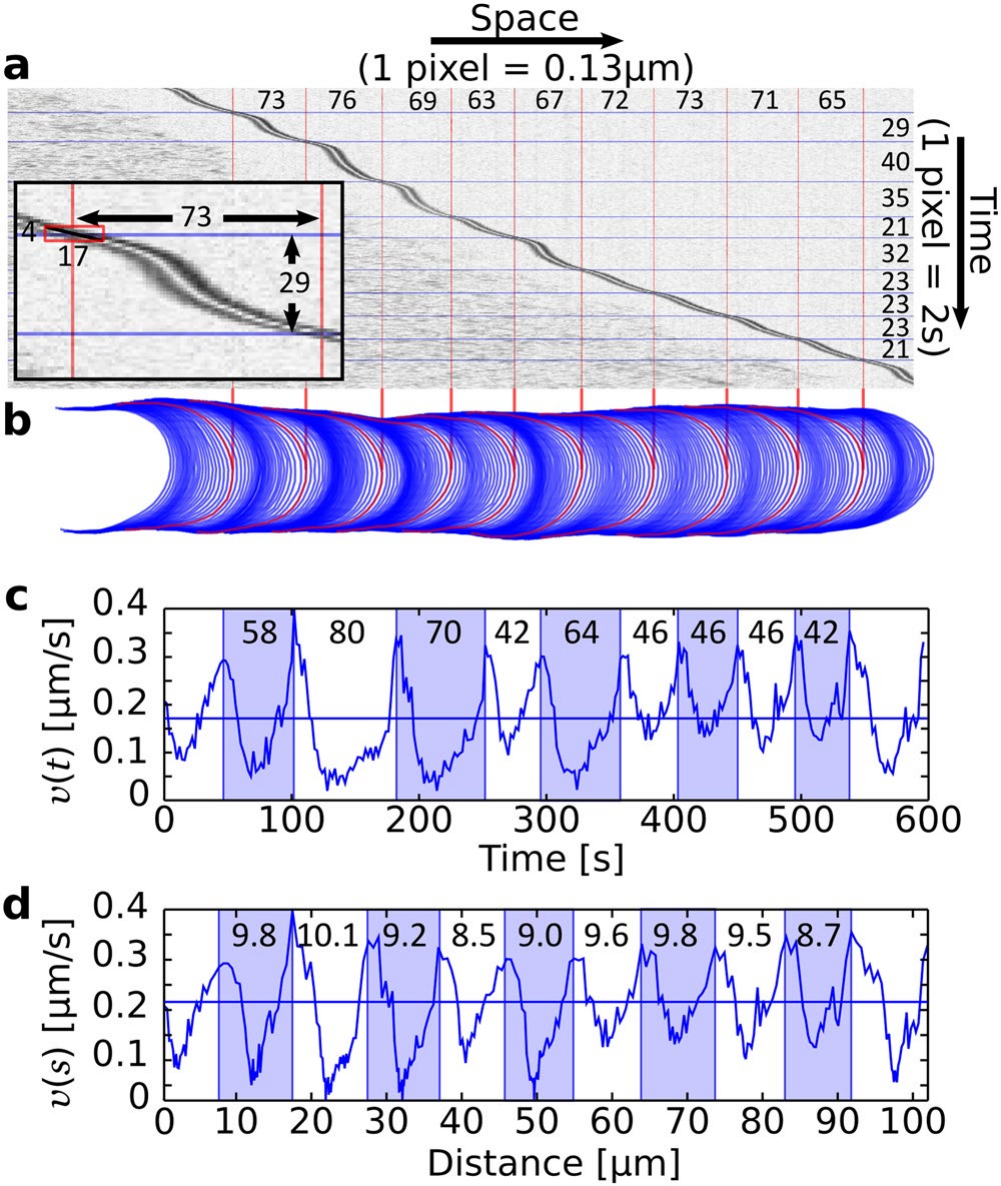
Precision estimates for the measurement of period and wavelength. (**a**) Kymograph of an oscillating lily pollen tube illustrating the relatively stable wavelength (CV(*λ*) = 6%) and fluctuating period (CV(*τ*) = 25%). The kymograph was prepared by adjoining a single row of pixels lifted from the growth axis of the cell at each time point (Methods). The total number of pixels is 974 for the spatial dimension and 300 for the temporal dimension. The wavelength (top) and period (right) for each cycle are indicated in pixel number. Inset: magnified view of the first cycle to reveal the individual pixels of the kymograph. (**b**) Contours of the cell captured at a 2 s time interval. (**c**) The velocity plotted as a function of time. The period of individual cycles is indicated in seconds. (**d**) The velocity plotted as a function of distance. The wavelength of individual cycles is indicated in microns.

As an alternative way to obtain quantitative information about the growth dynamics of pollen tubes, we prepared kymographs from image sequences using the function “reslice” of ImageJ. The “line” tool was used to select a 1-pixel-wide line centered on the growth axis of the cell. The stack of the time-lapse sequence was then resliced into a single image so that the rows represented the cell’s growth axis at successive time points. The kymographs were prepared either on the raw images captured by the camera or after using the “find edges” filter and inverting the histogram to enhance the contrast of the cell outline (Fig. 7a). The wavelength and period values extracted directly from the kymographs were nearly identical to those computed by tracking the cell outline in time and space (Fig. 7a), thus validating the two protocols.

### Time series analysis

The period of oscillation was defined as the time interval between successive growth rate maxima. The maximum of velocity is used to define the periods because it is much more precisely defined than the minimum or other points in the pulsatile cycle. The growth rate data were first smoothed and then Matlab’s findpeaks function was used to determine growth rate maxima. The best smoothed time series were obtained by combining the Savitzky-Golay filter and a moving average filter. The Savitzky-Golay filter performs, by means of least squares, a local polynomial regression of degree *k*, with at least *k* + 1 points equally spaced. The resulting polynomial is used to produce a smoother curve. The main advantage of this method is that it tends to preserve the maxima and minima^44^. To reduce the noise further, a moving average filter was applied. The average filter reduces the amplitude of the signal by leaves the period of oscillation unchanged.

For the time series analysis, we selected time windows with clear oscillations; that is, windows where the amplitude of oscillation was sufficient to identify isolated maxima and minima and where a given waveform was repeated at least two or three times. For most oscillatory cells (73%), this criterion excluded none of the information collected. In 27% of the cells, an average of 38% of the recording was removed for lack of a clear oscillatory pattern. The two groups of cells were first analysed separately and shown to have overlapping distributions of velocity, period, and wavelength. The two datasets were subsequently merged. In all cases, the velocity time series were truncated at the begining and/or the end of the series to eliminate incomplete oscillations.

### Error analysis

Two compounded errors affect the precision of our period and wavelength measurements: (*i*) the resolution used to measure space and time (resolution errors) and (*ii*) the choice of the start and end points for segmenting the time series into distinct oscillatory cycles (segmentation errors). The effect of these potential methodological errors can be tested graphically from kymographs prepared from our time-lapse sequences (Fig. 7a). In these image sequences, time and space are discretised with 1 pixel = 2 s for time and 1 pixel = 0.1339 *μ*m for space. To measure the wavelength and period, we chose the point of maximum velocity for the purpose of defining where each cycle starts and ends because these points are the easiest to pinpoint on the time series of the velocity.

The magnitude of the error on the selection of each endpoint is *ε*_*τ*_ = ±1 pixel = ±2 s. Therefore, in the case of the first cycle for the cell shown in Fig. 7a (inset), the precision of our measurement of period is *P*_*τ*_ = 2 × *ε*_*τ*_/*τ* = 2 pixel/29 pixel = 6.9% and the precision for the measurement of wavelength is *P*_*λ*_ = 2 × *ε*_*τ*_v*/*λ* = 2(17/4)/73 = 12%, where v* is the characteristic velocity at the start and end points of the oscillatory cycle (Fig. 7a (inset)). At the population level, the characteristic precision in the measurement of the period is ${P}_{\tau }=E[2\times {\varepsilon }_{\tau }/{\bar{\tau }}_{k}]=10 \%$. The characteristic velocity v* at the start and end of the oscillatory cycles can be calculated as a function of the mean velocity ${\bar{v}}_{k}$. We found that v* is on average: $(1.5\pm 0.3)\times {\bar{v}}_{k}$ (*n* = 159). Using this factor, the precision in the measurement of the wavelength is ${P}_{\lambda }=E[2\times {\varepsilon }_{\tau }{v}^{\ast }/{\bar{\lambda }}_{k}]=E[2\times 1.5{\varepsilon }_{\tau }{\bar{v}}_{k}/{\bar{\lambda }}_{k}]$ = $E[2\times 1.5{\varepsilon }_{\tau }/{\bar{\tau }}_{k}]=1.5{P}_{\tau }=15 \%$. Clearly, our measurement precision is better for the period than for the wavelength; thus excluding a potential methodological bias as an explanation for the greater fluctuations in the period.

### Choice of fitting function

We elected to fit the velocity using two in-phase sine functions, one with wavelength *λ* and a second with wavelength *λ*/2. Although other functional forms are possible and could perhaps give better fits, the functions we selected have the advantage of being the solutions to the simplest oscillatory system - the harmonic oscillator. Beyond the initial choice of the functional form, other assumptions on the fitting functions are based on the following observations:

i) positive and negative signs for parameter *a* correspond only to a phase shift in the solution by a factor *π* without any change to the waveform. Given this, we simply selected the positive quadrant.
ii) the sign of the parameter *b* corresponds to a left-right reflection of the waveform. Both positive and negative values of *b* are potentially useful for fitting the observed waveforms although a majority of waveforms calls for negative *b* values.
iii) the phase between the two sine functions must be zero so that equal distances are covered at high and low velocities (Fig. 5c).
iv) finally, for simplicity we included only the first two sine modes in the fitting function although many more could have been added to provide better fits.
